# Unmasking a Cerebral Arteriovenous Malformation Presenting as a Chronic Migraine-Type Headache at a District General Hospital

**DOI:** 10.7759/cureus.94936

**Published:** 2025-10-19

**Authors:** Kawser Ahmed, Anirban Deb Tanmoy, Hannah Aouad, Ashfaq Hamid

**Affiliations:** 1 Acute Internal Medicine, Northampton General Hospital NHS Trust, Northampton, GBR; 2 Internal Medicine, University Hospitals Dorset NHS Foundation Trust, Bournemouth, GBR; 3 Acute Medicine, Northampton General Hospital NHS Trust, Northampton, GBR

**Keywords:** cerebral arteriovenous malformation, computed tomography angiogram (cta), magnetic resonance angiography (mra), migraine-type headache, refractory headache

## Abstract

A female in her 50s was referred by her General Practitioner (GP) to our Same Day Emergency Care (SDEC) unit due to an unresponsive migraine attack. Associated features included nausea, photophobia, phonophobia, intermittent visual aura, and unintentional weight loss over the last two weeks. Arteriovenous malformations (AVMs) are rare vascular anomalies, with a prevalence of approximately 18 per 100,000 individuals. Although often asymptomatic, they may present atypically with migraine-like headaches, posing a diagnostic challenge.

Her full neurological assessment, including cranial nerves, cerebellar function, and motor and sensory systems, was unremarkable. However, neuroimaging revealed a large, unruptured, complex AVM in the left parieto-occipital region, associated with cerebral oedema and midline shift. Computed tomography angiography (CTA) and magnetic resonance angiography (MRA) confirmed a Spetzler-Martin Grade III AVM.

Her imaging was urgently discussed with the Neurosurgery Department at Oxford University Hospitals for further intervention, as our institution does not have a neurosurgery department being a district general hospital. The multidisciplinary team (MDT) recommended radiosurgery, and her symptoms resolved after the intervention.

This case illustrates the importance of neuroimaging in patients with refractory or unresponsive migraine, enabling early detection of serious intracranial pathology such as AVMs. It also highlights the value of timely specialist referral, inter-hospital collaboration, and patient-centred care in optimising outcomes.

## Introduction

Cerebral arteriovenous malformations (AVMs) are rare congenital vascular anomalies, usually diagnosed after catastrophic events such as intracerebral haemorrhage (ICH). The annual risk of spontaneous rupture is estimated at 2-4%, with significantly higher mortality [1]. However, as demonstrated in our patient, they may also present with progressive headaches, visual symptoms, aura, or seizures [2]. If an AVM is associated with cerebral oedema or midline shift, it may mimic a chronic primary headache [3]. The overall incidence and prevalence of AVMs are low, approximately 1 in 100,000 per year in unselected populations and 18 in 100,000 adults, respectively. The diagnosis of unruptured AVMs is challenging due to their non-specific symptoms. Therefore, careful selection of patients and neuroimaging techniques such as CT scan, magnetic resonance angiography (MRA), and CT angiography are the cornerstone of diagnosis and classification [4].

Assessing the risk of haemorrhage is crucial for determining the treatment strategy. There are different tools to assess haemorrhagic risk in AVMs, such as the Spetzler-Martin Grading Scale, Venous drainage, Age, Location, and Eloquence of adjacent brain tissue (VALE) score, and Race, Radiological evidence of prior silent haemorrhage, Eloquence of brain region involvement, and Deep location (R2Eed) [5]. While AVMs are well-documented, their presentation as refractory or unresponsive migraine-like headaches without focal neurological deficits remains a diagnostic challenge. Our case highlights the diagnostic value of early neuroimaging in patients with persistent or atypical migraine symptoms, even in the absence of red-flag headache features. The treatment relies on these risk stratifications, along with patient preference, to decide between conservative or interventional approaches (microsurgery, endovascular treatment, and radiosurgery) [6].

## Case presentation

A female in her 50s presented to the Same Day Emergency Care (SDEC) unit with a two-week history of a severe, progressively worsening headache. The headache, which was initially unilateral and pulsating, became constant, bilateral, and pressure-like, significantly affecting her activities of daily living (ADL). It was initially rated 9/10 in intensity and later reduced to 7/10. The headache was associated with photophobia, phonophobia, nausea, reduced appetite, and an unintentional weight loss of 2 kg over the same duration. She also reported intermittent neck stiffness and intermittent visual aura, particularly during severe attacks. Her migraines were previously well controlled with intermittent analgesia, and she was well known to the department for recurrent migraine attacks. However, this episode was noticeably different, more persistent and poorly responsive to high-dose over-the-counter analgesia (paracetamol 1 g four times daily and ibuprofen 600 mg three times daily). She denied fever, rash, seizures, or focal neurological symptoms. There was no recent trauma or illness. Her medical history was otherwise unremarkable, with no cerebrovascular or cardiovascular risk factors. She was a non-smoker and non-alcoholic. Family history was significant for a haemorrhagic stroke in her father in his 50s and neurofibromatosis type 1 in her aunt.

On examination, she was alert, oriented, and afebrile but distressed due to the ongoing headache. Vital signs were normal, and complete neurological and cardiovascular examinations, including fundoscopy and carotid auscultation, were unremarkable. Mild neck rigidity was noted, but there was no photophobia on examination and no Kernig’s or Brudzinski’s signs. Laboratory tests were unremarkable (Table 1).

**Table 1:** Basic laboratory tests performed prior to diagnosis. ALT: Alanine aminotransferase; ALP: Alkaline phosphatase; APTT: Activated partial thromboplastin time; eGFR: Estimated glomerular filtration rate; INR: International normalised ratio; WCC: White cell count.

Test	Result	Reference Range
Renal Function		
Urea	3.3 mmol/L	2.5-7.8 mmol/L
Creatinine	51 µmol/L	45-84 µmol/L
eGFR	>90 mL/min	>60 mL/min
Electrolytes		
Sodium	138 mmol/L	133-146 mmol/L
Potassium	4.0 mmol/L	3.5-5.3 mmol/L
Liver Function Tests		
Total Protein	70 g/L	60-80 g/L
Albumin	41 g/L	35-50 g/L
Bilirubin	7 µmol/L	0-21 µmol/L
ALT	13 IU/L	0-35 IU/L
ALP	54 IU/L	30-130 IU/L
Bone Profile		
Corrected Calcium	2.37 mmol/L	2.20-2.60 mmol/L
Inflammatory Markers		
CRP	1 mg/L	0-5 mg/L
Coagulation Profile		
INR	1	0.8-1.2
APTT	21 sec	22-30 sec
Full Blood Count		
Haemoglobin	142 g/L	120-150 g/L
WCC	6.8 × 10⁹/L	4.0-10.0 × 10⁹/L
Platelets	272 × 10⁹/L	150-400 × 10⁹/L

**Figure 1:**
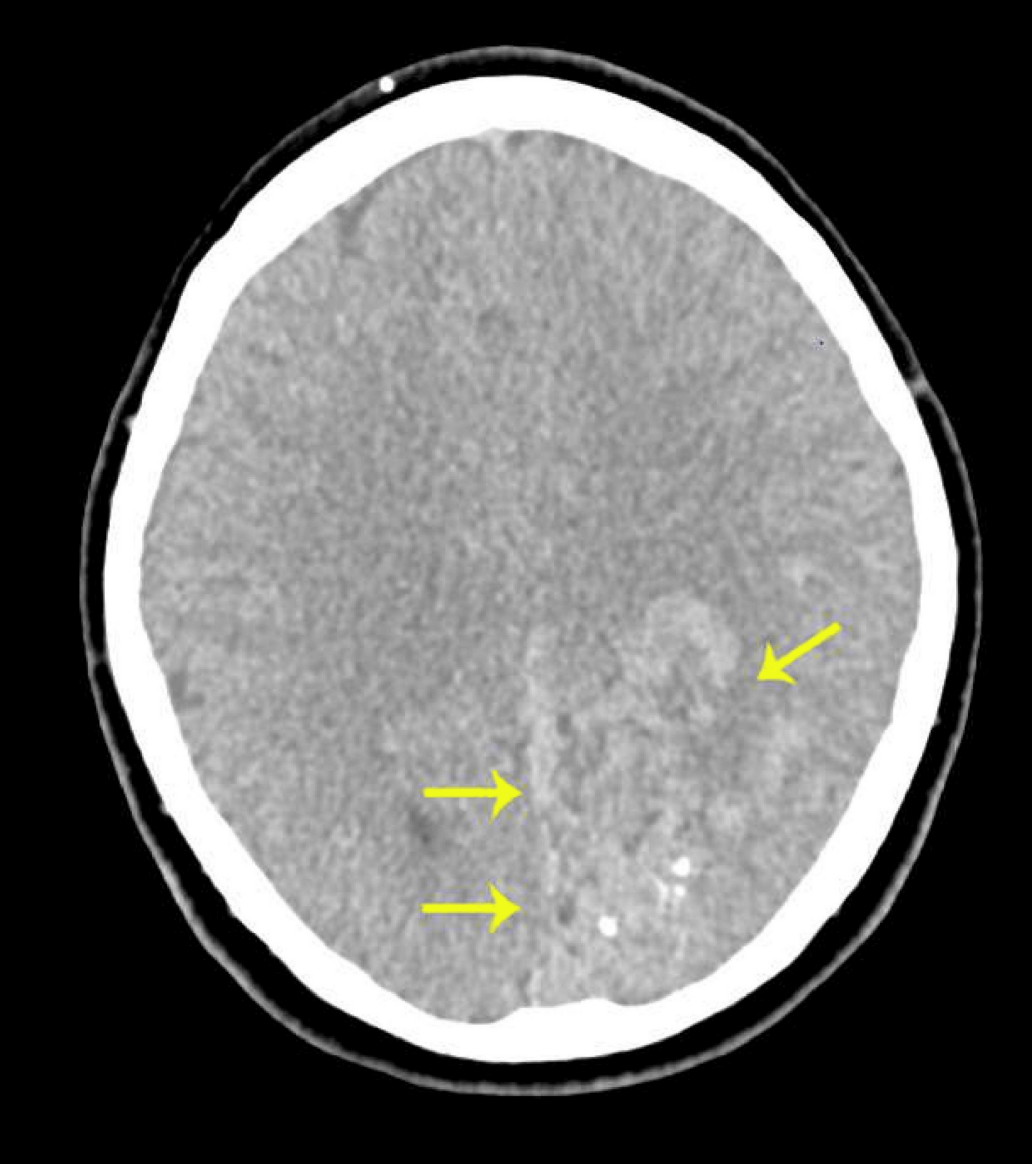
Non-contrast computed tomography (CT) of the head showing multiple curvilinear hyperdensities and punctate calcifications in the left parieto-occipital and temporal lobes. Surrounding vasogenic oedema and a 3 mm rightward midline shift are noted, without any evidence of acute haemorrhage.

**Figure 2:**
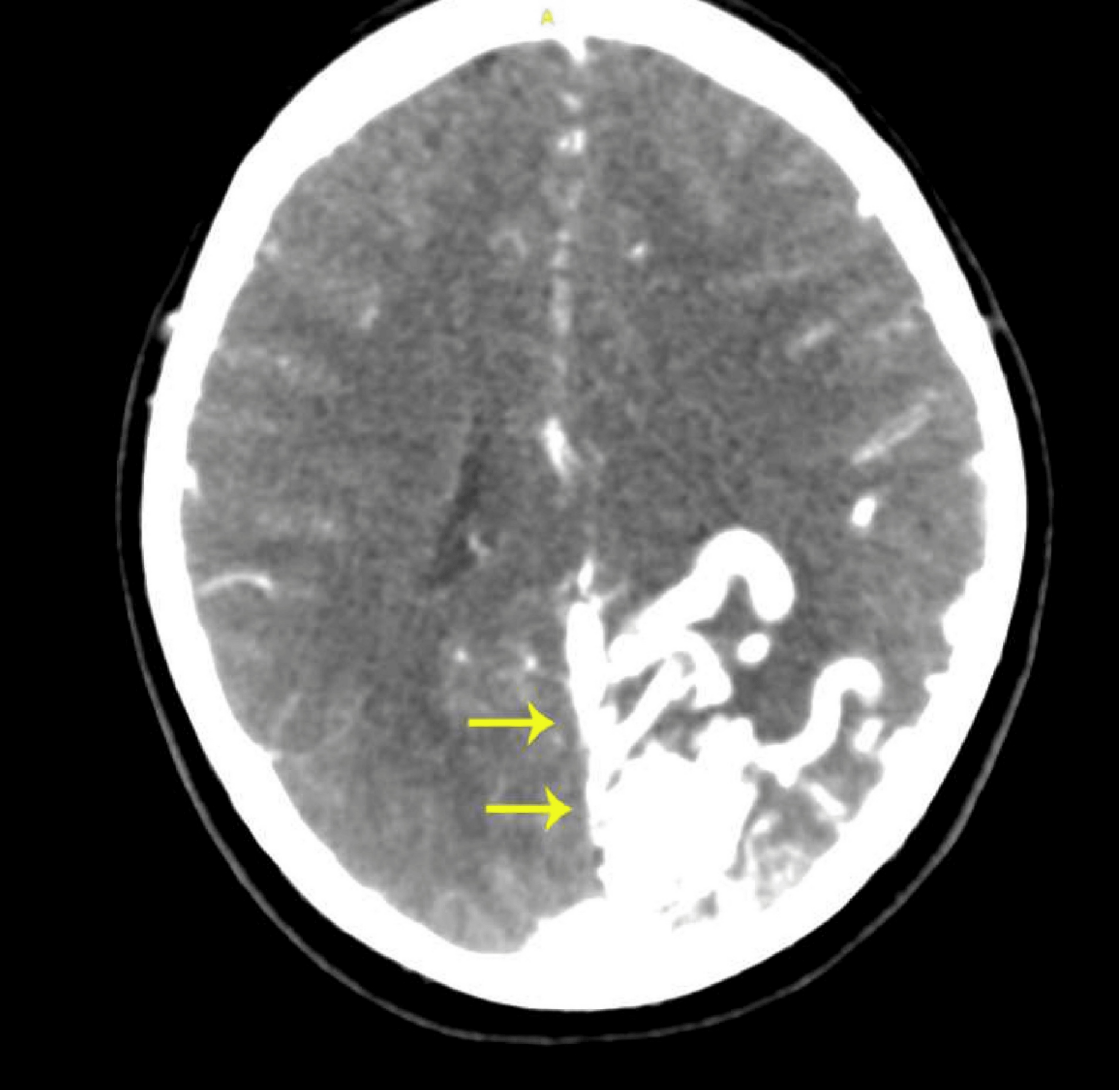
Computed tomography angiography (CTA) of the head showing a large, complex arteriovenous malformation measuring approximately 4 cm, supplied by branches of the left middle and posterior cerebral arteries, with venous drainage into the superior sagittal sinus.

**Figure 3:**
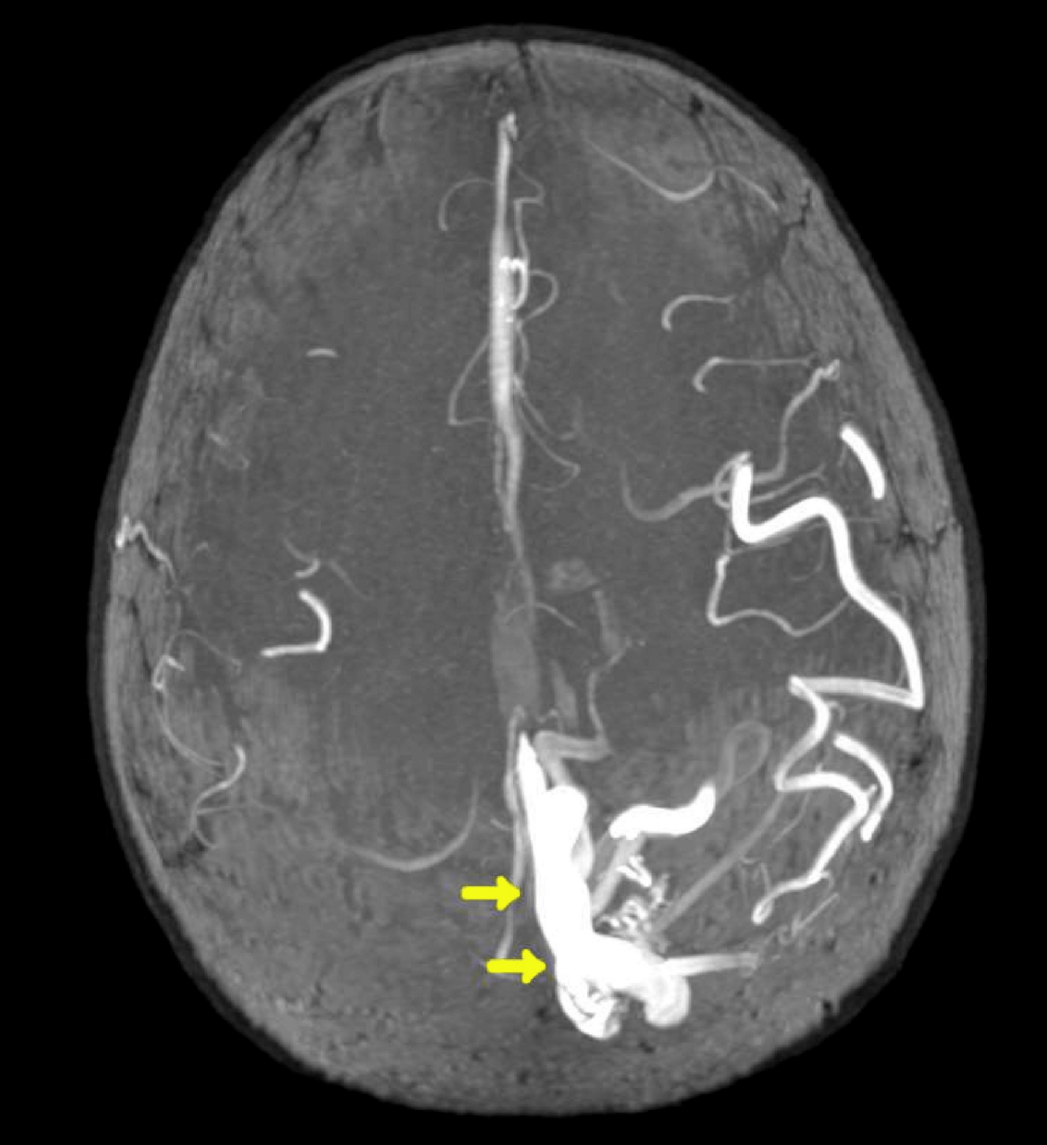
Magnetic resonance angiography (MRA) confirming the presence of a nidus-type arteriovenous malformation without evidence of acute or prior haemorrhage. Flow voids and surrounding oedema are noted, with no additional mass effect beyond the midline shift.

The initial differential diagnosis was an unresolving acute migraine attack or migraine with evolving features. Therefore, she received aspirin 900 mg in our SDEC immediately after the first assessment. Of note, she had self-administered sumatriptan 50 mg orally when she first experienced symptoms, as the medication was prescribed for her acute migraine treatment. She consumed 300 mg of sumatriptan over 24 hours without significant relief, as advised by her pharmacy. As the headache was unresponsive to high-dose analgesia and acute migraine therapy, the medical team broadened the differential diagnoses to include brain tumour, subacute intracerebral haemorrhage, and AVM. Hence, a non-contrast CT head was selected as the initial imaging modality due to its rapid availability and utility in identifying acute haemorrhage or structural brain lesions. Subsequently, CTA and MRA were performed, which confirmed the diagnosis. Neuroimaging revealed the following findings (Figures 1-3).

As the headache was significantly affecting her daily activities, and given the MRA and CTA classification of a Spetzler-Martin Grade III AVM as well as a positive family history, a shared decision was made to proceed with intervention at Oxford Neurosurgery. The procedure was uneventful, resulting in complete recovery of her migraine symptoms.

As she was transferred to another hospital, follow-up was conducted under the acute medicine virtual team, which revealed complete resolution of the headaches and visual symptoms. She was scheduled for subsequent follow-up at Oxford Neurosurgery at 3 and 6 months post-treatment. Repeat imaging at 6 months showed no evidence of rupture or progression of the disease. However, the imaging could not be retrieved as it was performed at another hospital. The patient informed our acute medicine team that she was planned for annual monitoring in the outpatient neurosurgery clinic at Oxford University Hospital.

## Discussion

The presented case is notable for the presence of an unruptured parieto-occipital AVM, initially misattributed to a migraine-like headache. The patient’s headache history spanned over 30 years, with fluctuating frequency and usually good response to standard migraine therapies. However, recent changes in headache characteristics and lack of response to standard treatment prompted neuroimaging. A patient-centred, judicious neurological work-up ultimately led to the correct diagnosis, followed by successful repair and a favourable outcome.

Headache is one of the most common presenting complaints in SDEC and Acute Medicine within the NHS in the United Kingdom [7]. In the vast majority of cases, the aetiology is benign and falls within the primary headache spectrum. Clinicians often rely primarily on clinical features and common red flags such as “the worst headache of life,” acute-onset thunderclap headache, neck stiffness, and focal neurological signs [8].

However, a common yet underappreciated diagnostic caveat lies in the tendency to downplay analgesic-unresponsive headaches, especially in patients with a long-standing diagnosis of migraine. These cases are frequently managed with advice to optimise analgesia, reassurance, and no further imaging. In this case, her diagnosis of migraine had been made decades earlier in primary care and was subsequently interpreted within that framework. Each encounter was treated as another migraine episode, and the patient was sent home with analgesia, leading to a form of diagnostic inertia. Unfortunately, this resulted in the underlying AVM, a potentially catastrophic lesion, being missed for years, metaphorically described as an “atom bomb” lying dormant in the brain [9].

While a definitive causal relationship between AVMs and headaches or seizures remains speculative, multiple lines of evidence support such an association in this case. The AVM was located in the parieto-occipital region, implicating involvement of the visual pathway [10]. This correlates with the patient’s intermittent visual auras and supports a causal relationship.

Occipital AVMs have been linked to both migraine-like headaches and seizures. The proposed pathophysiological mechanisms include vascular steal, cortical spreading depression, and perilesional oedema. In a retrospective observational study at the New York University Medical Centre, among a cohort of 70 patients with occipital AVMs, six patients with unruptured AVMs experienced both headache and seizures. The visual symptoms ranged from scintillating scotomas to blurring of vision [11]. One hypothesis suggests that AVMs can cause parenchymal oedema, as in our case, leading to prolonged headaches unresponsive to standard therapy [2].

## Conclusions

Neuroimaging plays a crucial role in evaluating refractory headaches that do not respond to conventional treatments, particularly in patients with chronic migraine. This approach is especially important because certain underlying conditions, such as AVMs in the parieto-occipital region, can mimic migraine symptoms and remain undetected for years. Clinicians should remain vigilant and consider neuroimaging when patients report changes in their migraine patterns, either in the form of unresponsiveness to standard therapy or the appearance of new symptoms, as these may indicate a more serious underlying condition. Early identification of such potentially treatable structural abnormalities can significantly alter patient management and improve outcomes.
